# Polybrominated diphenyl ether profiles in adipose tissues of breast cancer patients and their carcinogenic potential investigation based on network toxicology and molecular docking

**DOI:** 10.3389/fchem.2025.1630283

**Published:** 2025-09-24

**Authors:** Qihao Zhao, Xi Liu, Haoyi Chen, Yingming Jin, Qian Chen, Yiteng Huang, Lin Peng

**Affiliations:** 1 Central laboratory, Cancer Hospital of Shantou University Medical College, Shantou, Guangdong, China; 2 Shantou Key Laboratory of Precision Diagnosis and Treatment in Women’s Cancer, Shantou, China; 3 School of Public Health, Shantou University, Shantou, Guangdong, China; 4 Southern Medical University, Guangzhou, Guangdong, China; 5 Healthcare Center, First Affiliated Hospital of Shantou University Medical College, Shantou, Guangdong, China

**Keywords:** polybrominated diphenyl ethers, breast cancer, network toxicology, molecular docking, gas chromatograph-mass spectrometer

## Abstract

**Introduction:**

Existing epidemiological and experimental evidence have unveiled individual PBDE congeners facilitate the initiation of breast cancer. However, the comprehensive molecular mechanisms by which PBDE mixtures contribute to breast cancer pathogenesis remains poorly understood. This study aims to identify the PBDE congeners that preferentially accumulate in female adipose tissues and to intricate their interactions and key targets and molecular pathways implicated in breast cancer tumorigenesis.

**Materials and methods:**

Adipose tissue specimens were collected from 183 patients with breast cancer and 145 women with benign breast disease or non breast-related diseases. Adipose PBDEs concentrations were determined by gas chromatograph-mass spectrometer. The ChEMBL, STITCH, GeneCards, OMIM, TCGA-BRCA databases, as well as a protein-protein interaction (PPI) network, were utilized to identify the primary targets of PBDEs and their interactions. Molecular docking was performed using Autodock Vina to validate the binding affinities between chemicals and targets. Functional enrichment analysis was then performed based on Gene Ontology (GO) and Kyoto Encyclopedia of Genes and Genomes (KEGG) pathway analysis. Machine learning strategies were applied to refine core genes involved in pathogenesis of breast cancer.

**Results:**

BDE-47, BDE-138, BDE-153, BDE-183 and BDE-209 were recognized as the major PBDE congeners accumulated in adipose tissues. The top 20 candidate target genes were enriched for response to chemical stress, gland development, protein ligase binding, lipid and atherosclerosis and chemical carcinogenesis. The intersected genes and pathways between breast cancer and chemical carcinogenesis revealed significant associations with pathways in the PD-1/PD-L1 checkpoint and the HIF-1 signaling pathway. Machine learning strategies nominated CASP3, ESR1, MMP9, PARP1, and PPARG as crucial genes involved in breast cancer pathogenesis, exhibiting high-affinity binding to the major PBDE congeners.

**Conclusion:**

This integrative network study uncovers a mechanistic framwork linking adipose-accumulated PBDE mixtures to breast cancer pathogenesis. These findings provide insights for preventive and therapeutic interventions against PBDE-associated breast cancer.

## 1 Introduction

Polybrominated diphenyl ethers (PBDEs), a class of flame retardants extensively utilized in industrial products such as textiles, electronic appliances, furniture, and electronic devices, have been associated with a range of adverse health effects, including endocrine disruption, hepatotoxicity, neurotoxicity, reproductive toxicity, and potential carcinogenicity (Dong et al., 2023; Kostenko et al., 2024; Lan et al., 2024; Li et al., 2019; Wu et al., 2023; Yuan et al., 2021; Zhang et al., 2023). Due to their high lipid solubility and diverse exposure pathways, PBDEs can readily accumulate in adipose tissue, particularly in breast tissue (Author anonymous, 2017). PBDEs consist of 209 congeners, which are characterized by varying numbers and positions of bromine atoms on the aromatic ring. While bans and restrictions on PBDEs have been implemented in some countries and regions since 2003 (Programme, 2012), the lipophilic nature and resistance to degradation of PBDEs contribute to their ubiquitous presence in global environmental matrices, including soil, sediment, and air, as well as their detection in wildlife and human specimens (e.g., serum, urine, breast milk, umbilical cord blood, hair) (Alaee et al., 2003; de Wit, 2002; Li et al., 2018; Tang and Zhai, 2017; Xu et al., 2015).

Breast cancer remains the most commonly diagnosed cancer and the leading cause of cancer death among women in the world (Bray et al., 2024). Exposure to environmental chemicals, particularly persistent organic pollutants (POPs), have been linked to both the initiation and progression of breast cancer. Notably, the accumulation of POPs in breast adipose tissue has been more robustly associated with increased breast cancer incidence (Ennour-Idrissi et al., 2019). PBDEs, especially certain congeners, have been also identified as independent risk factors for breast cancer occurrence (He et al., 2018). The Endocrine-disrupting effects, DNA impairment and the inflammatory response have involeved in the primary mechanisms underlying PBDEs-induced cancer (Renzelli et al., 2023). However, the existing studies predominantly focused on the carcinogenic mechanisms of individual PBDE congeners rather than PBDEs mixtures (Dunnick et al., 2018; Lamkin et al., 2022), whereas the real-world human exposure scenarios typically involve complex mixtures of PBDEs.

Network toxicology is an emerging interdisciplinary field that integrates bioinformatics, systems biology, and chemical informatics to investigate the effects of chemicals on biological systems, by which how substances interfere with molecular networks thereby leading to cellular dysfunction and diseases could be elucidated (He et al., 2024). Molecular docking is a computational technique used to simulate interactions between environmental toxins and key proteins, facilitating insights into binding sites and conformations, thereby revealing the molecular underpinnings of pollutant toxicity (Trott and Olson, 2010). Critically, while studies on mechanism of individual PBDE congener *in vitro* or *in vivo* are abundant, the application to decipher the mechanisms of real-world human exposure to PBDE mixtures, based on congener profiles identified in actual patient tissues, remains unexplored. Herein, we hypothesize that exposure to environmentally relevant mixtures of major PBDE congeners contributes to breast cancer initiation by dysregulating specific pathways and key hub genes. To test the hypothesis and to elucidate the mixed carcinogenetic mechanisms of the major PBDE congeners (BDE-47, BDE-138, BDE-153, BDE-183, and BDE-209) in breast cancer patients, we employed an integrative approach combining epidemiological profiling, network toxicology and molecular docking.

## 2 Materials and methods

**FIGURE 1:**
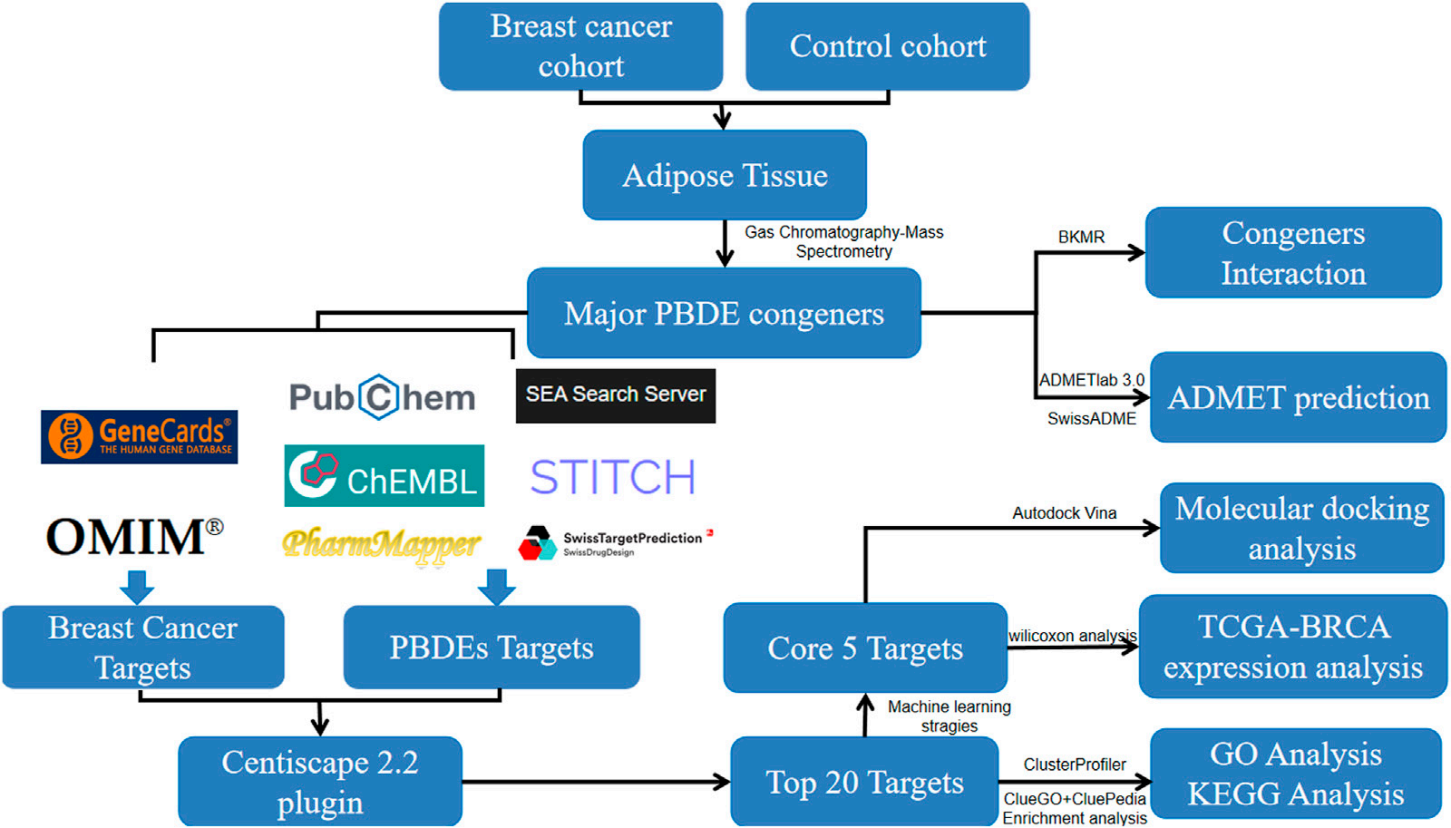
Integrative workflow of network toxicology and molecular docking analysis for this study.

The workflow was presented in Figure 1.

### 2.1 Acquisition of common PBDE congeners profiles in adipose tissues

A total of 183 patients with pathologically diagnosed Breast Cancer and 145 women with benign breast disease or non–breast-related diseases were retrospectively enrolled in our study from January 2014 to April 2015. Inclusion criteria of patients were as follows: (1) newly diagnosed with breast cancer; (2) born and residing in the eastern region of southern China. Two grams of human breast adipose tissue were collected and determined for PBDEs concentrations by an Agilent 7890A gas chromatograph coupled with an Agilent 5975C mass spectrometer (Agilent Technologies, USA). The procedures have previously been described in detail (He et al., 2017). To ensure a good linear relationship (r > 0.99), we established a set of multipoint standard curves (5-level for BDE-209 and 7-level for the other PBDE congeners). Clinical data, including age at diagnosis, menopause status, and family history of breast cancer, were retrospectively obtained from the hospital records and regularly interviews. This study was approved by the Human Ethics Committee of Shantou University Medical College (SUMC-05–2014), and all participants provided written informed consent after being fully informed of the study details and potential implications. The PBDEs profiles were visualized using radar charts. The five most abundant congeners were recoganized as the major compounds for further analysis.

### 2.2 Analysis of PBDE congeners mixture effect on breast cancer

Bayesian kernel machine regression (BKMR) model was utilized to estimate the mixed effects of the five major PBDE congeners on the occurrence of breast cancer, with adjusted for several variables known to be associated with breast cancer including age at diagnosis, menopausal status, and family history of breast cancer. When constructing the model with the bkmr (version 0.2.2) package, 10,000 iterations performed by Markov Chain Monte Carlo to ensure the convergence and stability of parameter estimation. The posterior inclusion probability (PIP) of each PBDE congener quantified its individual contribution to the model. The model evaluates the effect of the other factors maintained at the specific percentile (range from 0.1 to 0.9), as compared to the effect of their 50th percentile.

### 2.3 Acquisition of ADMET properties

To predict compound properties, ADMETlab 3.0 employed models, a novel methodology that was widely used to assess environmental hazards for their absorption, distribution, metabolism, excretion, and toxicity properties was performed by silico filtering using various machine learning methods (Zdrazil et al., 2024). To further validate the properties, we conducted the properties prediction of the congeners by SwissADME (Daina et al., 2017).

### 2.4 Acquisition of PBDEs targets

The structures of the major PBDEs congeners—BDE-47, BDE-138, BDE-153, BDE-183, and BDE-209—were retrieved from PubChem (Kim et al., 2023). The targets of these compounds were identified through databases including ChEMBL, STITCH, PharmMapper, PubChem, Similarity Ensemble Approach, and SwissTargetPrediction, which were specifically curated for *Homo sapiens* (Daina and Zoete, 2024; Keiser et al., 2007; Kim et al., 2023; Szklarczyk et al., 2015; Wang et al., 2017; Zdrazil et al., 2024). The target gained in all databases were accessed in October 2024. Then the names of the targets were standarized by the UniProt database (UniProt, 2024). The targets for each compound were then integrated and deduplicated.

### 2.5 Acquisition of disease-related targets

Breast cancer-related targets were retrieved from databases including Online Mendelian Inheritance in Man (OMIM) and GeneCards (last accessed in October 2024), retaining entries with relevance scores exceeding 10.0. The disease-related gene list was then intersected with PBDE-target genes. Subsequently, their overlap was visualized with a Venn diagram generated using the ggplot2 package.

### 2.6 Conduction of protein-protein interaction network

STRING was employed to identify the relationship among the intersected genes with an interaction score≥0.400 restricted to *H. sapiens* (Szklarczyk et al., 2015). Targets predicted by at least two distinct PBDE congeners were subsequently imported into Cytoscape 3.10.3 for protein-protein interaction (PPI) network visualization.

### 2.7 Candidate genes screening and functional enrichment analysis

To screen candidate genes from the Protein-Protein Interaction (PPI) network, we employed a methodological approach inspired by He et al., utilizing the CentiScape 2.2 plugin (He et al., 2024; Scardoni et al., 2009). In brief, we assessed centrality metrics including Degree, Betweenness, and Stress for each protein and standardized the ranks of these three centrality metrics collectively using Min-Max normalization. By reranking the standardized ranks in descending order, we selected the top 20 target genes. Functional enrichment analysis was performed to elucidate the biological functions and metabolic pathways of the top 20 target genes, utilizing ClusterProfiler and the ClueGO + CluePedia plugin (Bindea et al., 2009; Wu et al., 2021). Furthermore, Cytoscape software and ClueGo + CluePedia plugin were employed to establish the first neighbor pathways for a primary-tier interactome capable of uncovering latent yet potentially pivotal crosstalk among signaling cascades. Specifically, we first built a core set that contained all pathways and genes related to the top 20 genes annotated to the keywords “breast cancer” or “chemical carcinogenesis” (GO Biological Process, GO Molecular Function, GO Molecular Function and KEGG). CluePedia then retrieved every pathway that shared at least one gene or pathway with any member of this core set; these directly connected pathways or genes were defined as “the first neighbors”.

### 2.8 Analysis of core genes with machine learning strategies

Three machine learning strategies were employed to identify core targets among the top 20 genes: Least Absolute Shrinkage and Selection Operator (LASSO) analysis, Random Forest (RF) algorithm, and Support Vector Machine–Recursive Feature Elimination (SVM-RFE) algorithm (Hu and Szymczak, 2023; Kang et al., 2021; Sanz et al., 2018). In the LASSO analysis, the regression model was constructed with the glmnet package. We set “binominal” in “family” parameter and chose the optimal λ (lambda) value with its minimum value. For the SVM-RFE analysis, the e1071, kernlab, and caret packages were utilized to identify feature genes using the svmRadial model, with the doParallel package accelerating the computations. In the RF analysis, the randomForest package was employed to build a model with feature genes according to their importance among targets. Overall, the core targets were screened using the three machine learning strategies. The gene expression data used in this study were sourced from The Cancer Genome Atlas (TCGA) database from the Breast Invasive Carcinoma (BRCA) project, which was analyzed and visualized using ggplot2 (Wickham, 2016).

### 2.9 Molecular docking

The UniProtKB IDs of the top 20 genes, along with their common names, were retrieved from the UniProt database (UniProt, 2024). Corresponding human proteins of reviewed status were subsequently sourced from the Protein Data Bank (Supplementary Table 1), the protein structures were then retrieved from AlphaFold Protein Structure Database or predicted by AlphaFold 3 (Abramson et al., 2024; Varadi et al., 2024). The structures of the top 20 proteins were standardized by removing water molecules, adding hydrogen atoms. The grid box was set by PrankWeb selecting the rank 1 active position as the box center (Jakubec et al., 2022). The potential binding between compounds and proteins was analyzed using AutoDock Vina (Trott and Olson, 2010). Finally, the visualization of ligand-receptor binding was performed using Maestro Viewer, Schrödinger (Sankar et al., 2022). To rigorously validate our docking protocol, we utilized the agonist/antagonist annotated in Drugbank (Knox et al., 2024) to conduct molecular docking control analysis with the same AutoDock Vina parameters used for the 20 target proteins (Supplementary Table 1).

### 2.10 Validation of core genes

The validation of core genes involved differential mRNA expression analysis, survival analysis, and quantification of PBDEs in adipose tissue. We determined an mRNA expression profile that MCF7 cell expression was exposed to PBDEs (BDE-47, BDE-100, and BDE-153) in GSE111203 from the Gene Expression Omnibus (GEO) repository (Kanaya et al., 2019). Differential expression analysis compared to the dimethyl sulfoxide (DMSO) control group was conducted using independent samples t-tests for the PBDEs-exposed profile based on the result of tests of normality and homogeneity of variance test. Using data from the TCGA-BRCA database (accessed October 2024), the differential expression of five core genes (CASP3,ESR1,MMP9,PARP1,PPARG) were quantitatively analyze in breast cancer tissues compared to normal control tissues (Camp et al., 2004). Based on our previous study, we analyzed PBDEs content in human breast adipose tissue (He et al., 2018).

### 2.11 Statistical analysis

Statistical analyses were conducted using the ggplot2 package in R and IBM SPSS Statistics version 27.0.1.0 software (Team, 2024; Wickham, 2016). Gene expression values are represented as mean ± standard deviation (SD). According to the normal distribution distribution and homogeneity of variance of gene expression in GSE111203, significance was assessed using independent samples t-tests. Given the skewed distribution and heteroscedasticity of gene expression data from the TCGA-BRCA dataset, the Wilcoxon rank-sum test was employed. The optimal cutoff values for the expresion of core genes were determined with X-tile 3.6.1 software, as presented in Supplementary Figure S1. A p-value of less than 0.05 was considered statistically significant.

## 3 Results

### 3.1 Acquisition of major PBDE congeners in relation to breast cancer risk

**FIGURE 2:**
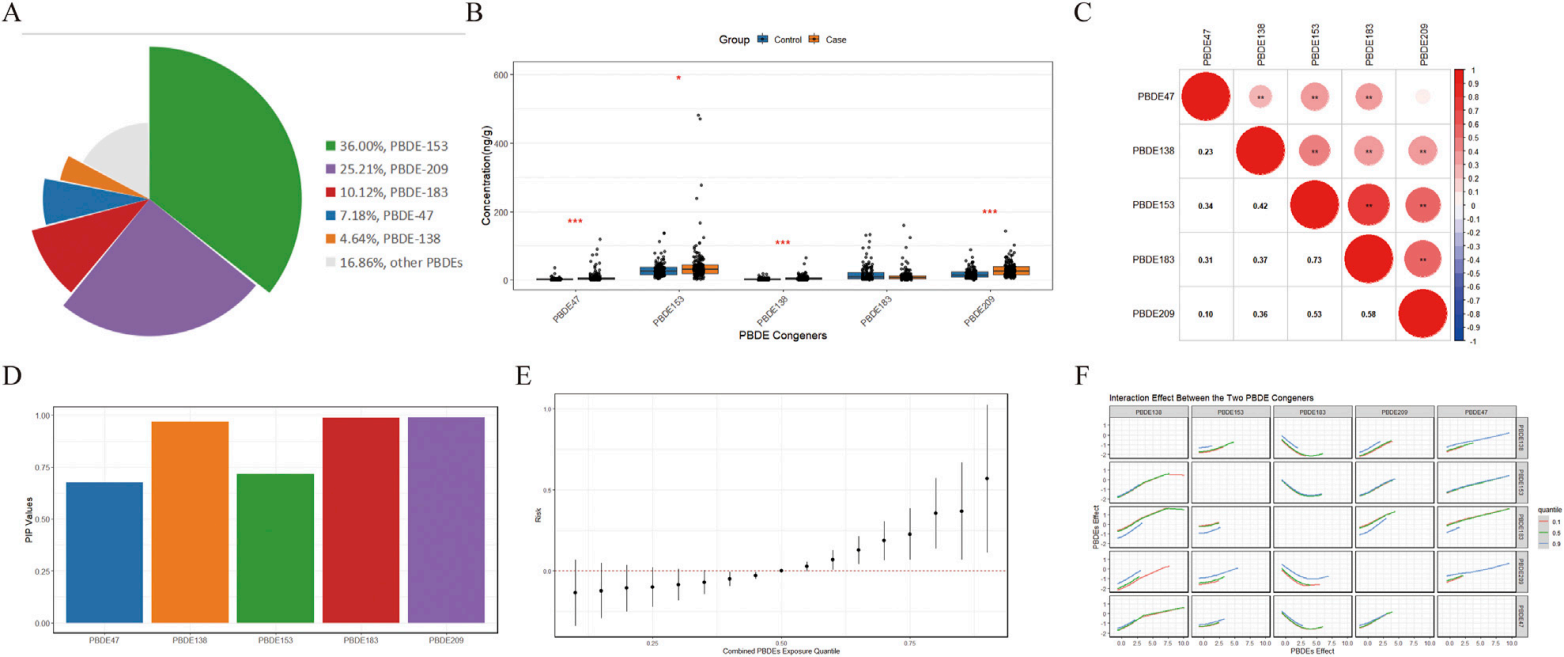
The common PBDE congeners identification and analysis of their effect on breast cancer. **(A)** PBDE congeners concentration profile in control group presents as radar chart. **(B)** Comparison of individual PBDE concentrations in cases and controls by Wilcoxon signed rank test. **(C)** Spearman correlation analysis of the major PBDE congeners. **(D)** PIPs value for each congener were presented in a bar chart. **(F)** The interaction analysis among the major PBDE congeners. The red dotted line represents the 50th percentile effect of PBDE congeners mixture on breast cancer. **(E)** Overall effect of the major PBDE congeners on breast cancer risk using the BKMR model, with adjusted for age at diagnosis, menopause status, as well as family history of breast cancer. **(F)** Interaction effect between each two PBDE congeners analyzed by BKMR model. * presents the p-value <0.05. ** presents the p-value <0.01. *** presents the p-value <0.001.

BDE-47, BDE-138, BDE-153, BDE-183, and BDE-209 were identified as the five most abundant PBDE congeners in case group (Figure 2A). The baseline characteristics of participants are listed in Table 1. BDE-47, BDE-138, BDE-153, and BDE-209 exhibited significantly different accumulation patterns between the case and control groups, while BDE-183 ranked among the top three PBDE congeners in both groups (Figure 2B). Consequently, BDE-47, BDE-138, BDE-153, BDE-183, and BDE-209 were identified as the major PBDE congeners for further study. To evaluate the correlations among the major PBDE congeners, Spearman correlation analysis was conducted, confirming strong correlations among them (Figure 2C). The major PBDE congeners play a role in the mixed effect (Figure 2D), with BDE-138 (PIPs = 0.969), BDE-183 (PIPs = 0.986), and BDE-209 (PIPs = 0.990) being the top three congeners that contribute the most to the mixed effect on breast cancer risk, followed by BDE-47 (PIPs = 0.676) and BDE-153 (PIPs = 0.716). Using the BKMR model, we observed a significant increase in breast cancer risk when all major PBDE congeners were fixed at or above their 55th percentile relative to the 50th percentile reference (Figure 2E). Notably, no significant interactions were found among congeners (Figure 2F), implying the absence of collinearity in the model.

**TABLE 1:** General characteristics and breast cancer risk factors for cases and controls.

Characteristics	Cases (*n* = 183)	Controls (*n* = 145)	*P*
Age (Mean ± SD, [range], years)	52.08 ± 9.77 [25–83]	43.57 ± 11.12 [24–68]	0.014189
PBDE-47 (median (IQR) ng/g)	3.86 (2.29–6.45)	2.55 (1.44–4.54)	<0.001
PBDE-138 (median (IQR) ng/g)	2.76 (1.22–7.10)	1.91 (1.01–3.94)	0.005
PBDE-153 (median (IQR) ng/g)	30.04 (18.03–45.15)	26.29 (16.96–38.36)	0.106
PBDE-183 (median (IQR) ng/g)	7.09 (4.52–12.58)	8.28 (4.11–18.71)	0.184
PBDE-209 (median (IQR) ng/g)	24.89 (14.01–37.97)	16.95 (10.85–25.90)	<0.001
Family of breast cancer history			
Yes	35	1	<0.001
No	148	144	
Menopausal status			
Premenopause	82	81	0.047
Postmenopause	101	64	
Marital status			
Yes	182	145	1.00
No	1	0	
Place of residence			
Shantou	113	102	0.411
Jieyang	35	20	
Chaozhou	31	21	
Other	4	2	
Breastfeeding			
Yes	174	123	0.002
No	9	22	
Number of children born			
0	5	0	0.10
1	39	37	
≥2	139	108	

Independent sample*t*-test was used for the continuous variables, and chi-square test or Fisher’s exact test was used for categorical data.

^a^ Since the non-normality of the data was confirmed by the Shapiro-Wilk test, we compared the sample median using the Mann-Whitney U test.

### 3.2 Intersection analysis of the co-target genes of PBDE congeners and breast cancer

**FIGURE 3:**
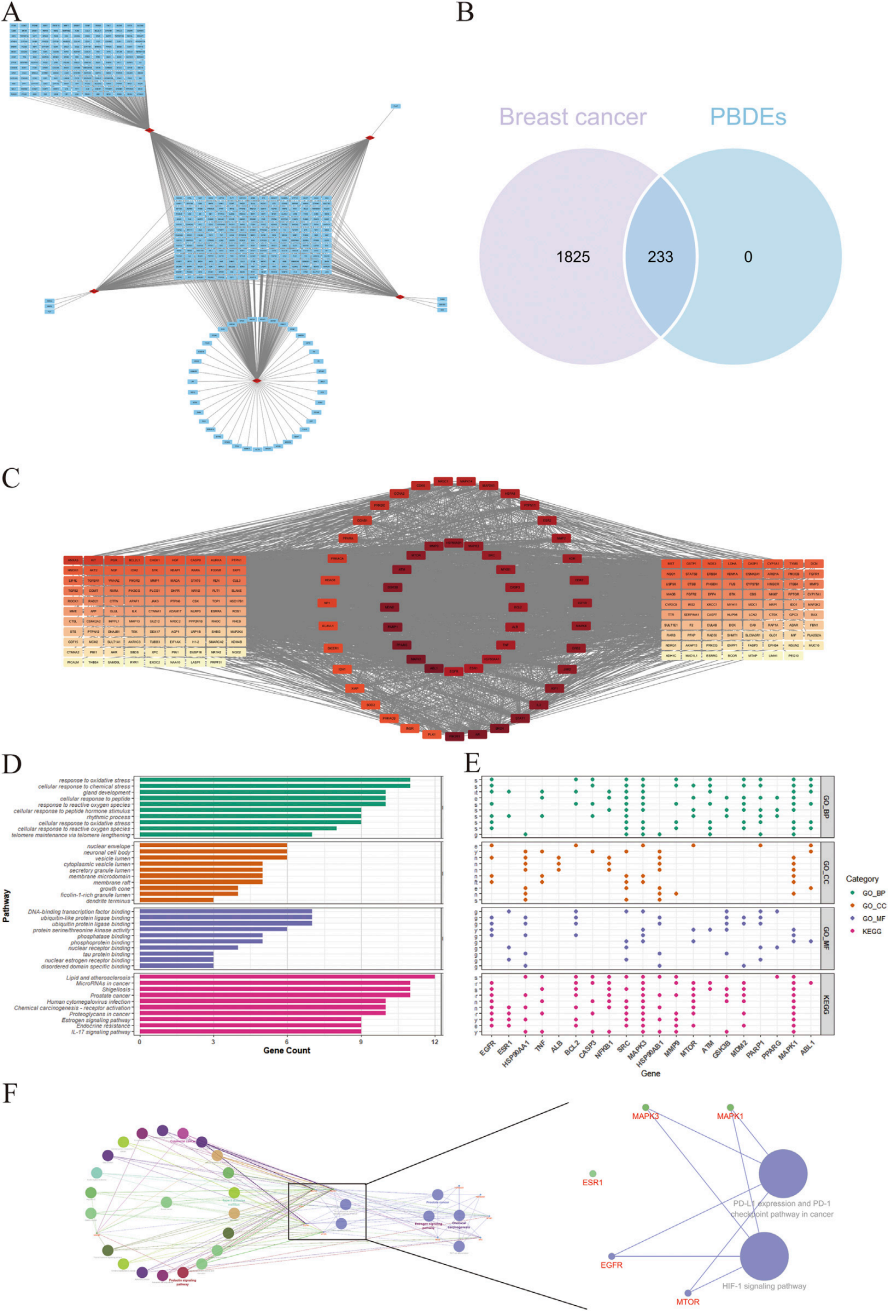
Candidate genes screening co-targeted by chemicals and breast cancer. **(A)** Relationship diagram between the five congeners and their target genes. The red rhombuses represent the major PBDE congeners, while the blue rectangles represent the genes predicted by databases. **(B)** The venn diagram shows the interested target genes intersected by PBDEs and breast cancer. **(C)** Centrality-based hierarchical visualization of key genes in a protein-protein interaction network. **(D)** The GO and KEGG enrichment analysis for the top 20 targets conducted by the ClusterProfiler and ggplot2. **(E)** The dotplot of the contributed genes in the pathways of GO and KEGG enrichment analysis. **(F)** The overview of the first neighbor pathways and genes of breast cancer and chemical carcinogenesis conducted by ClueGO plugin in Cytoscape software.

The chemical information for PBDEs, including their SMILES structures, molecular weights, and chemical formulas, is presented in Supplementary Table 2. By integrating and de-duplicating the target genes predicted for breast cancer from various databases, a total of 2,208 unique genes were retrieved. These genes are depicted as a grid in the center (Figure 3A), and 233 target genes predicted by more than two compounds are highlighted in the central grid. A total of 2058 target genes of breast cancer were identified through the databases. The intersection of breast cancer and PBDEs target genes result in 233 interested genes shown (Figure 3B). The 233 interested genes are probably contributed to PBDEs induced breast cancer. Based on that, the PPI network of the interested genes was presented (Figure 3C). Genes were organized in concentric circles if their betweenness, stress, and degree centrality values exceeded the average score. Higher ranks of the genes, indicating larger values, are depicted in red, while lower ranks are shown in yellow. The top 20 candidate target genes (Table 2) are displayed in the central concentric circle, suggesting their crucial role in breast cancer.

**TABLE 2:** Topological measurements of top 20 genes in the PPI networks, identified utilizing the CentiScape 2.2 plugin of Cytoscape.

Protein	Node	Betweenness	Degree	Stress	Rank
Epidermal growth factor receptor	EGFR	4893.902867	146	49906	1
Estrogen receptor	ESR1	3461.765416	126	43664	2
Heat shock protein HSP 90-alpha	HSP90AA1	2,134.546215	121	31726	3
Tumor necrosis factor	TNF	1846.435182	123	30034	4
Albumin	ALB	2088.173552	116	29002	5
Apoptosis regulator Bcl-2	BCL2	1204.545992	117	22582	6
Caspase-3	CASP3	1252.059671	112	22108	7
Nuclear factor NF-kappa-B p105 subunit	NFKB1	1293.387255	111	21226	8
Proto-oncogene tyrosine-protein kinase Src	SRC	1309.771054	107	20232	9
Mitogen-activated protein kinase 3	MAPK3	1122.746063	103	17982	10
Heat shock protein HSP 90-beta	HSP90AB1	1052.03426	102	19524	11
Matrix metalloproteinase-9	MMP9	1006.814487	98	17020	12
Serine/threonine-protein kinase mTOR	MTOR	930.4250484	93	15466	13
Serine-protein kinase ATM	ATM	878.8977161	84	15900	14
Glycogen synthase kinase-3 beta	GSK3B	790.4484594	91	14656	15
E3 ubiquitin-protein ligase Mdm2	MDM2	796.6072172	86	14280	16
Poly [ADP-ribose] polymerase 1	PARP1	789.5238713	79	15578	17
Peroxisome proliferator-activated receptor gamma	PPARG	731.2425848	83	12614	18
Mitogen-activated protein kinase 1	MAPK1	621.9825549	85	11946	19
Tyrosine-protein kinase ABL1	ABL1	955.0015405	65	13816	20

The detailed results of ADMET analysis using ADMETlab 3.0 and SwissADME are presented in Supplementary Table 3, which describe the properties of PBDEs. The symbol “+” indicates a low relationship between PBDE congeners and various properties, while “+++” indicates a strong relationship. PBDEs exhibit low gastrointestinal absorption and poor permeability in Madin-Darby Canine Kidney (MDCK) cells (less than 2 × 10^−6^ cm/s), indicating limited absorption potential. However, PBDEs show high bioactivity by effectively inhibiting P-glycoprotein and cytochrome P450 enzymes (CYP1A2, CYP2C19, and CYP3A4). They also demonstrate strong plasma protein binding (PPB >90%) and good blood−brain barrier (BBB) penetration (>90%), facilitating plasma protein binding and distribution. Most PBDEs have low plasma clearance (<5 mL/min/kg, except BDE-47 at 5.65 mL/min/kg) and short half-lives (1–8 h), suggesting their poor potential for excretion. Toxicologically, PBDEs were predicted to inhibit peroxisome proliferator-activated receptor gamma (PPARG), activate mitochondrial membrane potential, and affect steroid hormone receptor function (BDE-47 was predicted to activate estrogen receptor (ER), whereas other congeners act as receptor suppressors). Additionally, PBDEs are highly toxic to the heart, liver, skin, eyes, and respiratory system.

### 3.3 Network analysis of candidate genes in breast cancer-related enrichment results of GO and KEGG

The GO analysis (Figures 3D,E) demonstrated the biological processes including response to chemical stress, response to oxidative stress, and gland development, all of which are highly associated with chemical-induced breast cancer. In terms of cellular component enrichment, vesicle lumen and nuclear envelope were suggested as the potential target components. Within the molecular function enrichment analysis, protein ligase binding, including ubiquitin and ubiquitin-like binding, may play a crucial role in responding to chemically induced breast cancer. And KEGG enrichment analysis (Figures 3D,E) revealed pathways such as lipid metabolism and atherosclerosis, chemical carcinogenesis, endocrine resistance, and estrogen signaling pathway, which were enriched by the top 20 candidate target genes and are highly associated with PBDEs-induced breast cancer. Based on the enrichment analysis with ClueGO + CluePedia plugin in Cytoscape, we subsequently extracted the first neighbor pathways and genes that were related to breast cancer and chemical carcinogenesis (Figure 3F). PD-L1 expression and PD-1 checkpoint pathway in cancer and the HIF-1 signaling pathway were demonstrated to have connectivity between breast cancer and chemical carcinogenesis. Additionally, ESR1, EGFR, MAPK3, MTOR, and MAPK1 were identified to be related genes involved in.

### 3.4 The core targets screened by machine learning and their expression in TCGA-BRCA database

**FIGURE 4:**
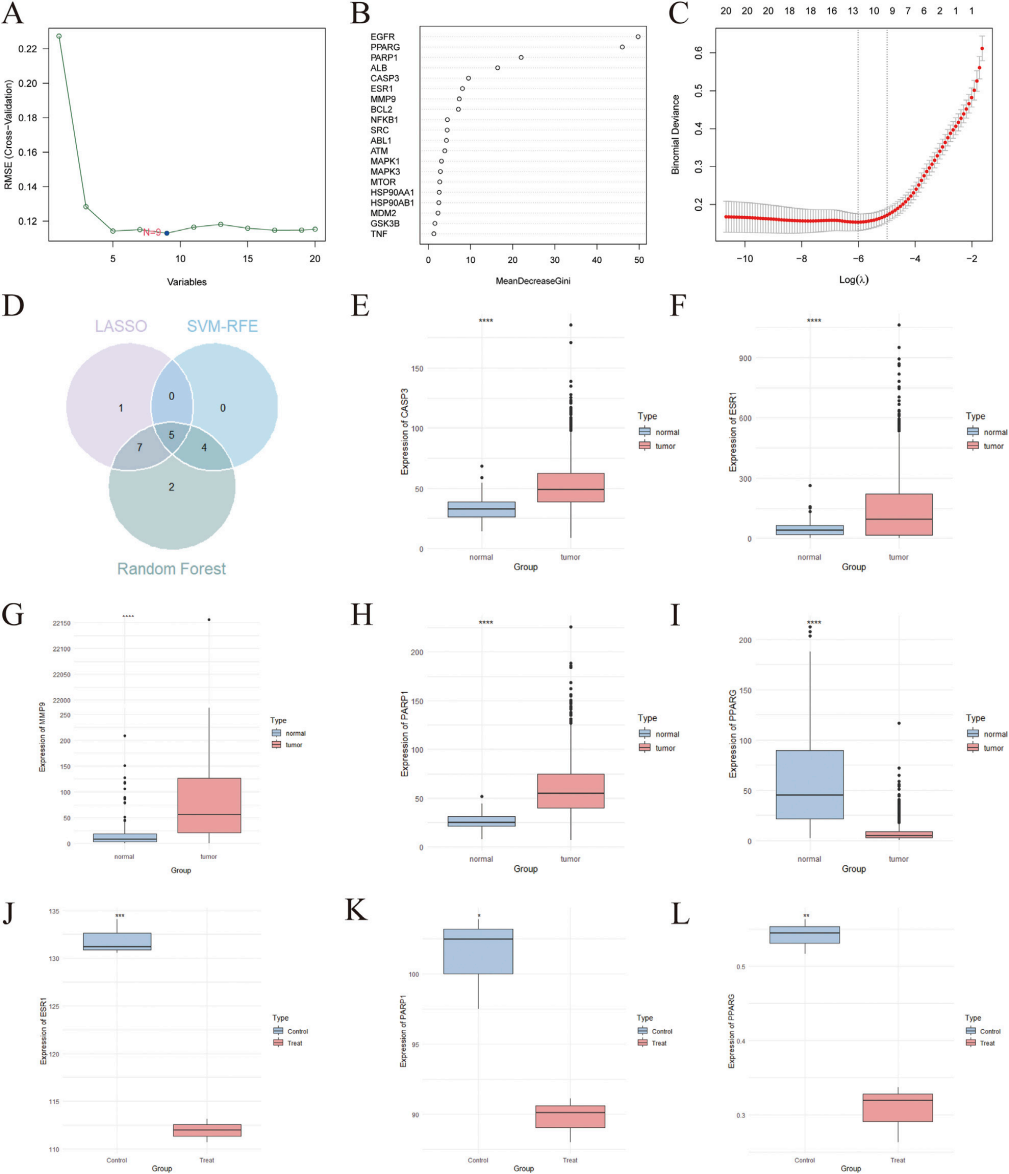
The core target genes identified by machine learning and TCGA-BRCA database. **(A)** Nine candidate core targets were identified by SVM-RFE machine learning algorithm. **(B)** The RF algorithm randomly selected 18 candidate core targets and ranked them with their importance among the targets. **(C)** At the lowest point of the curve, the number of genes were identified is thirteen constructed by LASSO regression model. **(D)** Veen diagram demonstrated the five core target for PBDEs-induced breast cancer which were intersected by the three machine learning algorithm strategies. The expression of key genes, CASP3 **(E)** ESR1 **(F)** MMP9 **(G)** PARP1 **(H)** and PPARG **(I)** in TCGA-BRCA database was shown as box plot. Expression analysis of ESR1 **(J)**, PARP1 **(K)** and PPARG **(L)** was visualized using box plots. **** presents the p-value of the expression analysis result <0.0001. *** presents the p-value of the expression analysis result <0.001. ** presents the p-value of the expression analysis result <0.01. * presents the p-value of the expression analysis result <0.05.

SVM-RFE, LASSO regression, and RF machine learning algorithms were employed to identify core target genes. The SVM-RFE algorithm pinpointed nine candidate core targets (Figure 4A). Subsequently, the RF algorithm was utilized to rank these genes based on their importance (Figure 4B). Additionally, the LASSO regression algorithm identified 13 candidate core targets, as shown in Figure 4C. The intersection of these gene sets, comprising five genes (CASP3, ESR1, MMP9, PARP1, and PPARG), was determined to be the core targets through a Venn diagram analysis (Figure 4D). Thereafter, mRNA expression analysis was conducted on the TCGA-BRCA database using the Wilcoxon test. The expression levels of the five core target genes were visualized as box plots (Figures 4E–I). Notably, these genes displayed markedly elevated or downregulated expression levels in tumor tissues, suggesting that their dysregulation may play a significant role in the pathogenesis of breast cancer.

### 3.5 Molecular docking of the top 20 candidate proteins

**FIGURE 5:**
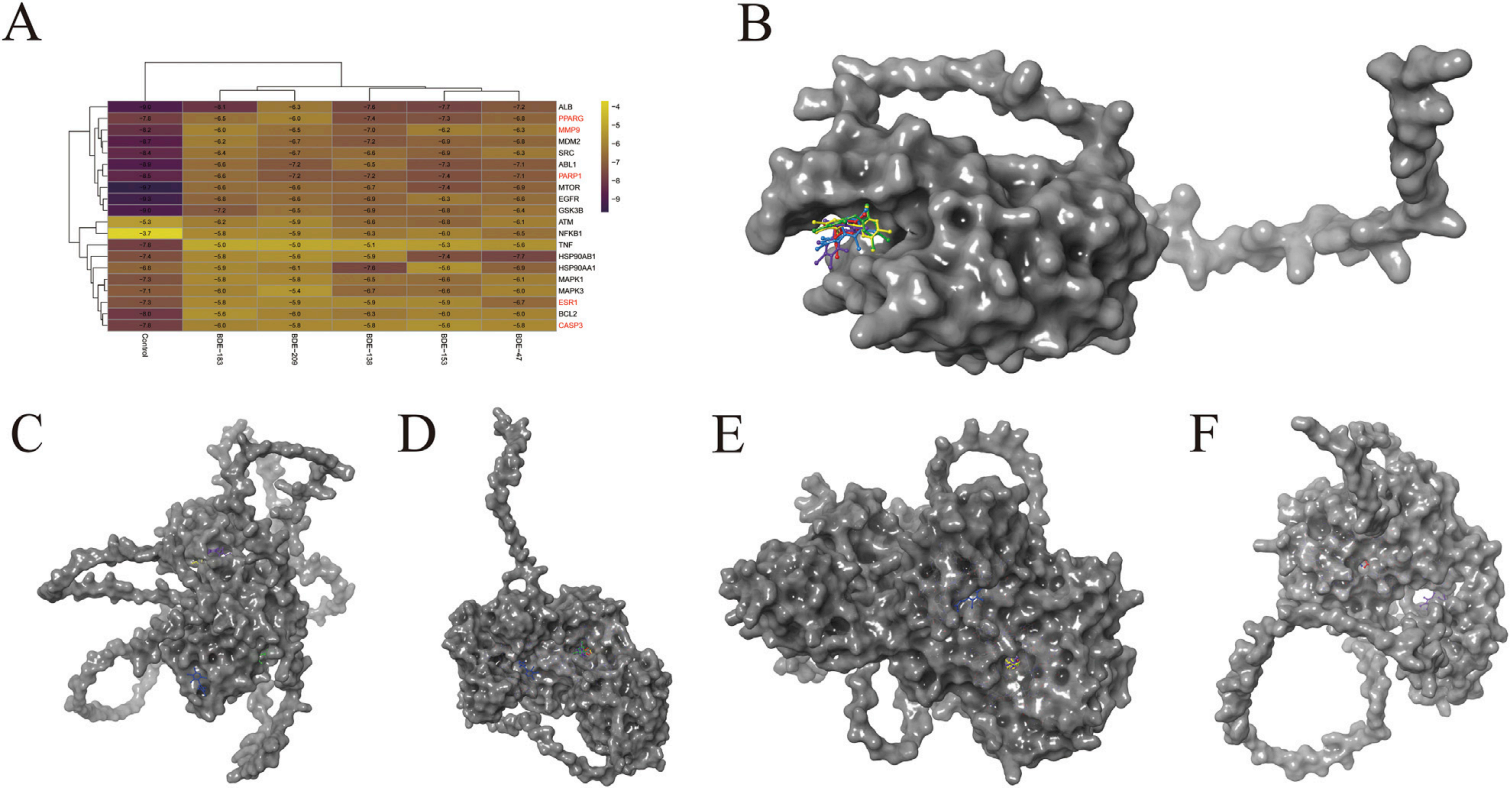
Molecular docking of the five core genes/controls with the major PBDEs. **(A)** The overview of the binding energy between the ligands and the proteins. CASP3 **(B)**, ESR1 **(C)**, MMP9 **(D)**, PARP1 **(E)**, and PPARG **(F)** were respectively binding with BDE-47 (red), BDE-138 (yellow), BDE-153 (green), BDE-183 (blue), and BDE-209 (violet).

Molecular docking simulations were performed between the top 20 candidate proteins and the major PBDE congeners. Binding energies between the 5 core proteins (controls, CASP3, ESR1, MMP9, PARP1, and PPARG) and each compound were visualized as heatplot (Figure 5A). Representative binding conformations are then depicted in Figures 5B–F. The red, yellow, green, blue, and violet ligands represent BDE-47, BDE-138, BDE-153, BDE-183, and BDE-209, respectively. The comprehensive docking parameters are provided in Supplementary Figures S2-S6. The halogen bonds, hydrogen bonds and hydrophobic interactions participate in the formation of the binding (Supplementary Figures S2-S6B, D, F, H, J), and several amino acid residues such as lysine (LYS), arginine (ARG), histidine (HIS) and threonine (THR) were got involved in the bindings between major PBDE congeners and the core proteins. Interestingly, the five compounds seem to have almost the same binding sites in each core protein except ESR1 (Figures 5B–F), implying the possibility of shared general toxicological mechanisms.

### 3.6 Validation of core genes and carcinogenesis effect of congeners

We further verified the expression levels of the core genes utilizing a dataset from the GEO. As a result, three genes were identified as differentially expressed between primary tumor specimens and normal tissue samples. Among them, ESR1 and PARP1 exhibited a significant increase within tumor tissues, while PPARG were significantly downregulated (Figures 4J–L; Supplementary Figures S1), implying that these genes may contribute to the pathogenesis of PBDEs-induced breast cancer.

## 4 Discussion

This study integrates network toxicology and molecular docking analyses to elucidate the potential mechanisms underlying the contribution of PBDEs to breast cancer initiation. Positive association between PBDEs mixture and breast cancer were found in BKMR model. Our findings highlight the complex interplay between PBDE congeners and key molecular pathways involved in breast carcinogenesis, providing novel insights into the toxicological basis of PBDEs-induced breast cancer.

Epidemic study revealed that PBDEs contribute to breast cancer as independent risk factors (Benoit et al., 2022; He et al., 2018) Confirmed by *in vitro* experiments, estrogen-like effect of PBDEs contribute to the proliferation of breast cancer cells (Renzelli et al., 2023). To further investigate the association between PBDEs mixture exposure and breast cancer risk, we employed the BKMR model for assessing mixed pollutant effects. The analysis revealed a positive correlation trend between PBDEs mixture exposure and breast cancer initiation. By constructing exposure-response surfaces through kernel functions, the BKMR model effectively addressed potential estimation biases inherent in traditional statistical methods, which often fail to fully account for collinearity issues among compounds and between pollutants and other risk factors in analyzing complex mixture exposure effects.

The results of the ADMET analysis identified potential health risks associated with PBDEs, including organ toxicity, metabolic interference, and accumulation in the body. The results revealed that PBDE congeners share several common characteristics, suggesting that additive or synergistic effects may occur when these substances are present in mixtures. In special, the findings of PBDE congeners exhibiting low absorption but high bioavailability in humans, implying that even exposure to low doses can induce biotoxicity. Additionally, we found that these congeners have a strong ability to bind proteins but are difficult to excrete, which is consistent with their widespread distribution and accumulation in the body. The current results also showed that all the substances have potential to cross the blood-brain barrier, suggesting their potential to reach the central nervous system. PBDEs that remain covalently or tightly bound to plasma proteins are indeed too large to traverse the intact BBB. However, consistent with our finding, an ADMETlab *in silico* assessment (Qu et al., 2025) indicated that PBDE-47 is highly lipophilic (logP ≈ 6–7) and has a low polar surface area, implying a high probability of passive diffusion across the BBB. Although no direct BBB transport experiment was performed, the subsequent network toxicology and transcriptomic analyses revealed significant dysregulation of neuroinflammation-, ferroptosis- and cell-cycle-related genes in human neural progenitor and neuron-like PC12 cells, providing indirect evidence that PBDE-47 can accumulate in the brain and exert neurotoxicity. This finding aligns with the neurotoxicity of PBDEs indicated in previous studies (Dong et al., 2023; Li et al., 2019). In the perspecive of toxicological pathways, the carcinogenesis and the genotoxicity of tumors may be due to the activation of steroid hormone receptors and changes in mitochondrial membrane potential, which is consistent with previous *in vitro* studies of PBDEs (Chen et al., 2022; Kanaya et al., 2019; Tian et al., 2024; Zhang et al., 2016). Previous studies have reported associations between PBDEs exposure and multi-organ toxicity, but there are limited studies on eye corrosion, irritation, and skin sensitization, providing new directions for future research (Dong et al., 2023; Kostenko et al., 2024; Wu et al., 2023; Yuan et al., 2021; Zhang et al., 2023). Based on ADMET analysis, our study indicated that these substances exhibit multiple adverse effects, including respiratory toxicity, hepatotoxicity, nephrotoxicity, and genotoxicity, besides eye corrosion and irritation, skin sensitization. The toxicological metabolic pathways analysis showed that PBDEs had low absorption but high bioavailability in human body, which may explain the findings that PBDEs inhibit P-glycoprotein and cytochrome P450 enzymes, consisting with our analysis (Li et al., 2017; Yu et al., 2021).

Oxidative stress is a well-acknowledged toxicological mode of effect for PBDEs (Jiang et al., 2024). In human embryonic stem cells, BDE-209 upregulated the expression of oxidative stress-related genes HIF1a and HIF2a (Du et al., 2016). With BDE-209 exposure, HIF1a expression were also increased in *sparus aurata* fibroblast cell line (Du et al., 2016). Notably, HIF-1 has been shown to drive breast tumorigenesis via Wnt/β-catenin pathway activation (Liu et al., 2021). Consistant with these observations, our network toxicology analysis identifies the HIF-1 pathway as a pivotal mediator of PBDEs-induced breast carcinogenesis. The PD-1/PD-L1 checkpoint pathway also emerged as a central pathway in PBDE-induced breast cancer. Although endocrine disruptor compounds such as bisphenol A, di-ethylhexyl-phthalate, dibutyl phthalate and 4-tert-octylphenol have been shown to modulate human macrophage responses (Couleau et al., 2015), our work is, to the best of our knowledge, the first to implicate PBDEs in tumor immune evasion circuitry. Rigorous experimental studies are now warranted to determine whether PBDEs exposure compromises immune surveillance and to underlying mechanisms.

In the current study, we indicated five core genes, including CASP3, ESR1, MMP9, PARP1, and PPARG, contribute to the pathogenesis of breast cancer. CASP3 (caspase-3), serving as an executor of apoptosis and the critical protein in pyroptosis, has been closely associated with tumor reproliferation, and the status of PR and HER2 in breast cancer (Rodriguez-Ruiz et al., 2020; Yang et al., 2018). Recent evidence indicated that Caspase-3 promotes oncogene-induced malignant transformation in mammalian cells via EndoG-dependent Src-STAT3 phosphorylation (Zhu et al., 2024). Moreover, Caspase-3 was found to orchestrate cytoprotective autophagy in human breast cancer cells subjected to starvation or proteasome inhibition (Samarasekera et al., 2025). These findings suggest the potential of caspase-3 in tumor recurrence. While exposure to PBDEs, such as BDE-47, BDE-153, and BDE-209, has been indicated to induce upregulation of caspase 3 in hepatocytes, mouse nerve cells, macrophages and neurons (Dong et al., 2024; McDermott et al., 2024; Meng et al., 2020; Wang and Dai, 2022), the role of caspase 3 in PBDEs-induced breast cancer initiation remains to be clarified.

MMP9 is a matrix metalloproteinase that allows for the invasion and metastasis of tumor cells via decomposition of extracellular matrix (ECM) components and basement membrane (BM). MMP9 overexpression in tumor cells has been associated with poor survival, larger tumor size, lymph node metastasis, distant metastasis, higher clinical stage, and histological grade in patients with breast cancer (Jiang and Li, 2021). MMP9 has been reported to exhibit elevated expression levels following exposure to BDE-209 and BDE-47 in melanoma and human neuroblastoma (Silva Filho et al., 2022; Tian et al., 2016). And the dysregulation of MMP9 expression was associated with the risk of breast cancer (Dofara et al., 2020). PBDEs exposure upregulates MMP9 expression, which may contribute to the breast cancer initiation. However, limit studies have focused on the mechanisms by which MMP9 induces breast cancer, indicating the necessity for further studies.

Previous studies demonstrated that exposore to BDE-209 and BDE-47 could trigger increased protein levels of cleaved PARP in hippocampus neuron (Li et al., 2019; Sun et al., 2017). The activation or the upregulation of PARP plays a role in single-strand DNA damage repair, and PARP1, the isoenzyme of PARP, repairs over 99% single-strand DNA damage (Ndlovu et al., 2024). PARP proteins, mainly located in nucleus, may be involved in signaling cascades, response to intracellular stress, apoptosis, mitochondrial function and energy metabolism in breast cancer (De et al., 2025), suggesting PBDEs-inducing breast cancer initiation may be synergistic with multiple causes.

Breast cancer cells, despite their reliance on an independent energy metabolism, still depend on mitochondria for DNA replication. The initiation of breast cancer and the emergence of drug resistance are influenced by mitochondrial-related genes, such as PPARG and ESR1 (Strillacci et al., 2022; Xu et al., 2025). Interestingly, our ADMET analysis found that PBDE is associated with mitochondrial membrane potential, suggesting breast cancer initiation induced by PBDEs may result from the regulation of mitochondrial regulation by crucial targets.

ESR1 primarily regulates gene transcription by binding to estrogen (Gui et al., 2025). PBDEs exposure, especially BDE-47, exhibits estrogen-like effects and activates estrogen receptor signaling pathway, aligning with our ADMET and KEGG analysis (Kanaya et al., 2019; Li et al., 2013). In ER-positive breast cancer, the activation of ESR1 bypasses the G1-S checkpoint to promote the tumor growth (Marra et al., 2023). Additionally, ESR1 mutations are more common in metastatic breast cancer, leading to constitutive activation of the estrogen receptor, which contribute to the proliferation and drug resistance of cancer cell (De Marchi et al., 2024). The findings suggest that ESR1 may contribute to the initiation of PBDEs-induced breast cancer.

The expression of PPARG is involved in adipocyte differentiation and metabolism, probably resulting in PBDEs toxicological effects. Exposed to BDE-47, BDE-99, and BDE-153, the upregulation of PPARG influence the lipid metabolism in mice adipose tissue or human adipose tissue (Liu et al., 2022; Liu et al., 2023; Wen et al., 2019), which may contribute to the different distribution of PBDEs on adipose and serum (Renzelli et al., 2023). In breast and gastric cancer, PPARG could inhibit tumor initiation and growth through regulating Wnt/β-catenin signaling pathway (Wang et al., 2024). The findings suggest that PPARG may result in the different distribution of PBDEs and the initiation of PBDEs-inducing breast cancer. In our analysis, the different expression pattern may result from the cell type, suggesting further experiment to validate.

Additionally, we explore the binding potential of the 5 crucial targets utilizing molecular docking analysis. The binding energy results of PBDE congeners with proteins were all below −5 kcal/mol, indicating the robust binding potential (Gao et al., 2025). The high-affinity binding of PBDEs to targets may regulate them through competitive inhibition (Chen et al., 2016) and conformational transition (Wang et al., 2013), affecting the initiation of PBDEs-inducing breast cancer. The binding of PBDEs to proteins may disrupt the biological function. Several amino acid residues such as LYS, ARG, HIS and THR were potential binding target residues of major PBDE congeners, which consist with the findings from other previous studies (Chen et al., 2023; Li et al., 2022; Wang et al., 2025; Xu et al., 2024).

Originally, we sought to link PBDE mixture burden with clinical stage within our cohort and to examine whether the identified core genes exhibited stage-dependent expression in TCGA-BRCA dataset. However, no significant relationships were detected (Supplementary Figures S7). These null findings align with our earlier report (Xie et al., 2023), where none of the five PBDE congeners included in the current study correlated with either clinical stage or TNM grade. Consequently, we cautiously infer that exposure to BDE-47, BDE-138, BDE-153, BDE-183 and BDE-209 preferentially influence breast-cancer initiation than subsequent progression.

Compared with traditional network toxicological analysis, our study identified research subjects based on epidemiological profiles of the predominant PBDE congeners that accumulate in female adipose tissue and subsequently validated crucial target genes in the TCGA database and GEO dataset, thereby enhancing clinical translatability. Through comprehensive bioinformatics and molecular docking analyses, we underscore a potential mechanism by which PBDEs contribute to the molecular etiology of breast cancer, by altering critical genes, cellular functions and pathways. The multiple databases and machine learning strategies partly avoid the potential false positives from single algorithm or machine learning strategy and reduce the time and cost associated with traditional animal experiments. However, several limitations should be warrant consideration. First, the TCGA cohort data lacks information on PBDE levels in breast adipose tissue, which limits our ability to draw definitive associations between PBDE exposure and target genes expression. Second, although BMI, smoking, alcohol consumption, and diet are well established risk factors for breast cancer, they were excluded from covariate set in the BKMR model because over 40% of control participants had missing data. This unavoidable omission may bias the exposure–outcome association. Third, while the silico analyses are comprehensive, the absence of experimental validation for identified target genes limits the mechanistic claims. Further research utilizing *in vitro* and *in vivo* models is imperative to solidify the causative relationship between PBDEs exposure and abnormal regulation of key genes and pathways involved in breast cancer carcinogenesis. For example, qRT-PCR and Western blotting could be employed to quantify transcript- and protein-level alterations of core genes in PBDE-treated breast cancer cell lines or patient-derived tumor organoids. Moreover, orthotopic xenograft models subjected to PBDE exposure will serve as an *in vivo* validation platform, integrating single-cell sequencing to elucidate PBDE-induced reprogramming of the tumor microenvironment. In addition, CRISPR/Cas9-based gene editing is available for functionally interrogating the role of each identified gene in PBDE-driven breast carcinogenesis.

## 5 Conclusion

This study integrates macro-level and micro-level analytical approaches to identify critical pathways and targets that influence the initiation of breast cancer with PBDEs exposure. Given the potential impact of key target expression changes and specific binding interactions, strategies to reduce PBDE exposure, such as the development of environmentally friendly flame retardants and the implementation of stricter regulatory guidelines, are urgently needed. The limitation of PBDE exposure may reduce breast cancer risk, increase sensitivity of chemical therapy, and complement existing treatments.

## Data Availability

The datasets GSE111203 for this study can be found in the Gene Expression Omnibus [https://www.ncbi.nlm.nih.gov/geo/query/acc.cgi?acc=GSE111203]. The datasets TCGA-BRCA for this study can be found in the National Cancer Institute Genomic Data Commons Data Portal [https://portal.gdc.cancer.gov/]. Data will be available on request due to the patient privacy.

## Supplementary material

SUPPLEMENTARY FIGURE S1 The optimal cutoff values for the survival analysis groups in terms of CASP3 **(A)** ESR1 **(B)** MMP9 **(C)** PARP1 **(D)** and PPARG **(E)** were selected by X-tile 3.6.1 software.

SUPPLEMENTARY FIGURE S2 Molecular docking analysis of CASP3 visualized in 3D and 2D. The red, yellow, green, blue, and violet ligands represent BDE-47 **(A,B)** BDE-138 **(C,D)** BDE-153 **(E,F)** BDE-183 **(G,H)** and BDE-209 **(I,J)** respectively. The legend presents the form of the ligand and receptor bond in 2D.

SUPPLEMENTARY FIGURE S3 Molecular docking analysis of ESR1 visualized in 3D and 2D. The red, yellow, green, blue, and violet ligands represent BDE-47 **(A,B)** BDE-138 **(C,D)** BDE-153 **(E,F)** BDE-183 **(G,H)** and BDE-209 **(I,J)** respectively. The legend presents the form of the ligand and receptor bond in 2D.

SUPPLEMENTARY FIGURE S4 Molecular docking analysis of MMP9 visualized in 3D and 2D. The red, yellow, green, blue, and violet ligands represent BDE-47 **(A,B)** BDE-138 **(C,D)** BDE-153 **(E,F)** BDE-183 **(G,H)** and BDE-209 **(I,J)** respectively.

SUPPLEMENTARY FIGURE S5 Molecular docking analysis of PARP1 visualized in 3D and 2D. The red, yellow, green, blue, and violet ligands represent BDE-47 **(A,B)** BDE-138 **(C,D)** BDE-153 **(E,F)** BDE-183 **(G,H)** and BDE-209 **(I,J)** respectively. The legend presents the form of the ligand and receptor bond in 2D.

SUPPLEMENTARY FIGURE S6 Molecular docking analysis of PPARG visualized in 3D and 2D. The red, yellow, green, blue, and violet ligands represent BDE-47 **(A,B)** BDE-138 **(C,D)** BDE-153 **(E,F)** BDE-183 **(G,H)** and BDE-209 **(I,J)** respectively. The legend presents the form of the ligand and receptor bond in 2D.

SUPPLEMENTARY FIGURE S7 Association between clinical stage and both breast-adipose PBDE mixture burden and core-gene expression in breast cancer patients. **(A)** Overall effect of the PBDE mixture on the probability of advancing clinical stage evaluated by BKMR analysis within our cohort, adjusted for age at diagnosis, menopause status, BMI and family history of breast cancer. **(B)** Wilcoxon signed rank test for differential expression of each core gene between early-stage (Stage Ⅰ/Ⅱ) and late -stage (Stage Ⅲ/Ⅳ) tumors in the TCGA-BRCA dataset.
