# Supplementary material for: Polybrominated diphenyl ether profiles in adipose tissues of breast cancer patients and their carcinogenic potential investigation based on network toxicology and molecular docking
Source: Front Chem. 2025 Sep 24;13:1630283. doi: 10.3389/fchem.2025.1630283 (PMC12505496; doi:10.3389/fchem.2025.1630283)
Supplement: Supplementary file 2 [file Table1.docx]

Supplementary Table 1: The Uniport ID and their molecular docking control Drugbank ID of top 20 proteins.

| Proteins | Uniport ID | Drugbank ID |
| --- | --- | --- |
| ABL | P00519 | DB08043 |
| ALB | P02768 | DB08496 |
| ATM | Q13315 | DB02289 |
| BCL2 | P10415 | DB17023 |
| CASP3 | P42574 | DB08498 |
| EGFR | P00533 | DB15327 |
| ESR1 | P03372 | DB07567 |
| GSK3B | P49841 | DB08073 |
| HSP90AA1 | P07900 | DB07317 |
| HSP90AB1 | P08238 | DB08293 |
| MAPK1 | P28482 | DB07788 |
| MAPK3 | P27361 | DB04604 |
| MDM2 | Q00987 | DB17549 |
| MMP9 | P14780 | DB07246 |
| MTOR | P42345 | DB12180 |
| NFKB1 | P19838 | DB01822 |
| PARP1 | P09874 | DB04010 |
| PPARG | P37231 | DB08760 |
| SRC | P12931 | DB08564 |
| TNF | P01375 | DB07145 |

Supplementary Table 2: Chemical information mentioned of five most abundant PBDE congeners.

| PBDE congeners | Chemical formula | SMILE structure | MW(g/mol) |
| --- | --- | --- | --- |
| PBDE-47 | C12H6Br4O | C1=CC(=C(C=C1Br)Br)OC2=C(C=C(C=C2)Br)Br | 485.79 |
| PBDE-138 | C12H4Br6O | C1=CC(=C(C(=C1OC2=CC(=C(C=C2Br)Br)Br)Br)Br)Br | 643.60 |
| PBDE-153 | C12H4Br6O | C1=C(C(=CC(=C1Br)Br)Br)OC2=CC(=C(C=C2Br)Br)Br | 643.60 |
| PBDE-183 | C12H3Br7O | C1=C(C(=CC(=C1Br)Br)Br)OC2=C(C(=C(C=C2Br)Br)Br)Br | 722.50 |
| PBDE-209 | C12Br10O | C1(=C(C(=C(C(=C1Br)Br)Br)Br)Br)OC2=C(C(=C(C(=C2Br)Br)Br)Br)Br | 959.20 |

Supplementary Table 3: Drug-likeness and ADMET prediction result of the five most abundant PBDE congeners.

| Description | BDE-47 | BDE-138 | BDE-153 | BDE-183 | BDE-209 |
| --- | --- | --- | --- | --- | --- |
| Physicochemical properties | | | | | |
| Molecular formula | C12H6Br4O | C12H4Br6O | C12H4Br6O | C12H3Br7O | C12Br10O |
| Molecular weight (g/mol) | 485.79 | 643.58 | 643.58 | 722.48 | 959.17 |
| Hydrogen bond acceptors count | 1 | 1 | 1 | 1 | 1 |
| Hydrogen bond donors count | 0 | 0 | 0 | 0 | 0 |
| Rotatable bonds count | 2 | 2 | 2 | 2 | 2 |
| Topological polar surface area | 9.23 | 9.23 | 9.23 | 9.23 | 9.23 |
| Pharmacokinetic parameters | | | | | |
| Absorption | | | | | |
| GI absorption | Low | Low | Low | Low | Low |
| Caco-2 permeability | -5.037512596 | -5.029705172 | -5.046100919 | -5.022965101 | -5.006536132 |
| MDCK permeability | -4.780227231 | -4.810874555 | -4.835915943 | -4.817867966 | -4.653249956 |
| F30% | + | + | + | + | + |
| Distribution | | | | | |
| PPB | 98.06% | 98.00% | 98.20% | 97.70% | 96.53% |
| VD | 0.5838266799569479 | 0.5902162415326659 | 0.6061996434386976 | 0.4785221640436075 | 0.43753851206875094 |
| BBB | +++ | +++ | +++ | +++ | +++ |
| Fu | 1.07% | 1.14% | 0.80% | 1.39% | 2.53% |
| Metabolism | | | | | |
| CYP1A2 inhibitor | +++ | +++ | +++ | +++ | +++ |
| CYP2C19 inhibitor | +++ | +++ | +++ | +++ | +++ |
| CYP3A4 inhibitor | + | +++ | +++ | +++ | +++ |
| CYP3A4 substrate | + | + | + | + | + |
| P-gp inhibitor | +++ | +++ | +++ | +++ | + |
| P-gp substrate | + | + | + | + | + |
| Excretion | | | | | |
| CL | 5.647115932016238 | 1.9001278293534694 | 1.947456828331073 | 1.9577748650848914 | 1.1824309188548652 |
| T½ | 1.0259579018541578 | 2.800210562917501 | 2.6742762219316214 | 2.5434291084145606 | 3.157382126155241 |
| Toxicity | | | | | |
| hERG blockers | +++ | +++ | +++ | +++ | +++ |
| H-HT (hepatotoxicity) | + | + | + | + | + |
| DILI (Drug induced liver injury) | +++ | +++ | +++ | +++ | +++ |
| Skin sensitization | +++ | +++ | +++ | +++ | +++ |
| Eye –corrosion/irritation | +++ | +++ | +++ | +++ | +++ |
| Respiratory toxicity | ++ | ++ | ++ | ++ | ++ |
| Toxicity pathways | | | | | |
| NR-AR | ++ | +++ | +++ | +++ | +++ |
| NR-ER | +++ | + | + | + | + |
| NR-PPAR-gamma | + | + | + | + | + |
| SR-MMP | +++ | +++ | +++ | +++ | +++ |
| SR-p53 | + | ++ | ++ | ++ | + |
| Toxicophore rules | | | | | |
| Acute toxicity rule | 1 | 1 | 1 | 1 | 1 |
| Genotoxic-carcinogenicity rule | 0 | 0 | 0 | 0 | 0 |
| Non genotoxic carcinogenicity rule | 0 | 0 | 0 | 0 | 0 |
| Skin sensitization rule | 0 | 0 | 0 | 0 | 0 |
| Non-biodegradable rule | 0 | 0 | 0 | 0 | 0 |
| SURE-CHEMBL rule | 0 | 1 | 1 | 2 | 2 |
| FAF-drugs4 rule | 0 | 0 | 0 | 0 | 0 |

Here ++ consider as poor, while +++ present the worst case. + presents the predict value range from 0-0.3. ++ presents the predict value range from 0.3 - 0.7. ++ presents the predict value range from 0.7-1.0.
