# Supplementary figures and images for: Polybrominated diphenyl ether profiles in adipose tissues of breast cancer patients and their carcinogenic potential investigation based on network toxicology and molecular docking
Source: Front Chem. 2025 Sep 24;13:1630283. doi: 10.3389/fchem.2025.1630283 (PMC12505496; doi:10.3389/fchem.2025.1630283)

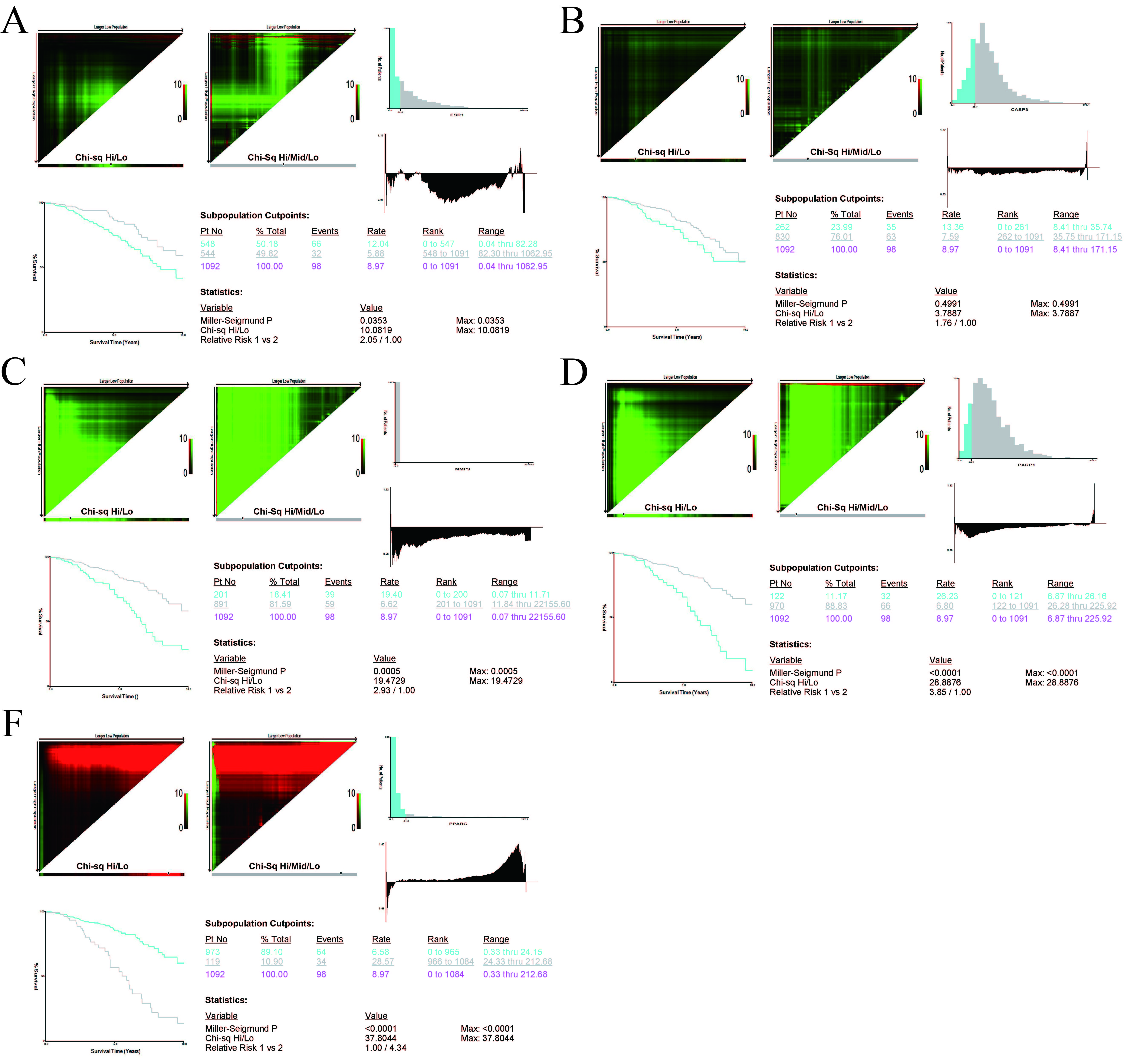

Supplement: Supplementary file 3 [file Image1.jpeg]

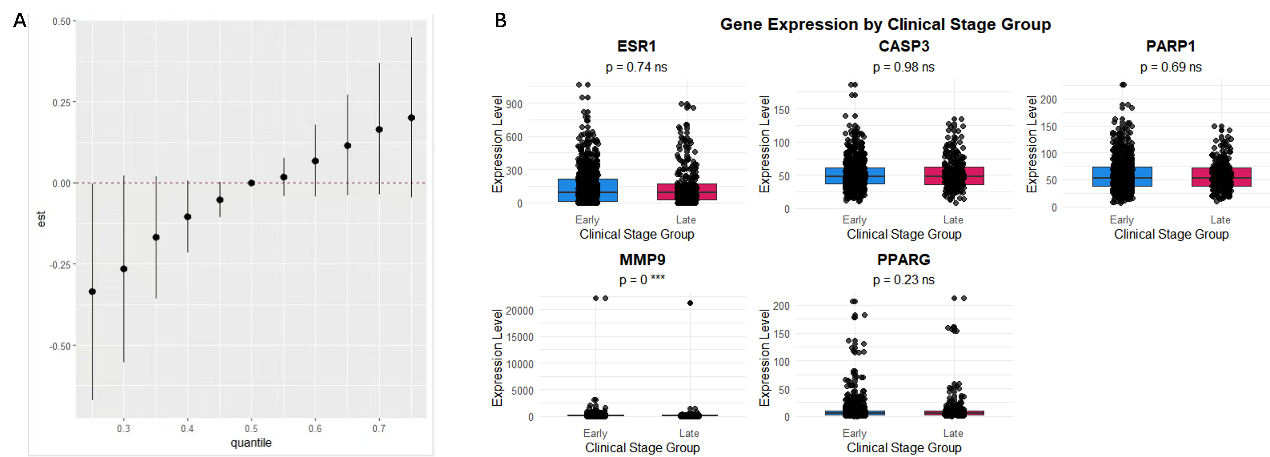

Supplement: Supplementary file 7 [file Image7.tif]
